# The effect of Israeli acute paralysis virus infection on honey bee brood care behavior

**DOI:** 10.1038/s41598-023-50585-4

**Published:** 2024-01-10

**Authors:** Lincoln N. Taylor, Adam G. Dolezal

**Affiliations:** Department of Entomology, University of Illinois Urbana-Champaign, Urbana, IL 61801 USA

**Keywords:** Entomology, Animal behaviour

## Abstract

To protect themselves from communicable diseases, social insects utilize social immunity—behavioral, physiological, and organizational means to combat disease transmission and severity. Within a honey bee colony, larvae are visited thousands of times by nurse bees, representing a prime environment for pathogen transmission. We investigated a potential social immune response to Israeli acute paralysis virus (IAPV) infection in brood care, testing the hypotheses that bees will respond with behaviors that result in reduced brood care, or that infection results in elevated brood care as a virus-driven mechanism to increase transmission. We tested for group-level effects by comparing three different social environments in which 0%, 50%, or 100% of nurse bees were experimentally infected with IAPV. We investigated individual-level effects by comparing exposed bees to unexposed bees within the mixed-exposure treatment group. We found no evidence for a social immune response at the group level; however, individually, exposed bees interacted with the larva more frequently than their unexposed nestmates. While this could increase virus transmission from adults to larvae, it could also represent a hygienic response to increase grooming when an infection is detected. Together, our findings underline the complexity of disease dynamics in complex social animal systems.

## Introduction

Organisms that live in social groups experience many benefits to their fitness and survival. For example, social animals have additional defenses against predation, improved foraging success, increased access to potential mates, and in some species cooperative care of offspring^1^. However, group living is also inherently linked to an increased risk of infectious disease due to high concentrations of susceptible hosts^2^, frequent close contacts^2,3^, and often high intragroup relatedness^2,4^. To combat this, social organisms supplement the immunological and cellular processes that compose an individual’s immune response with group-level adaptations for reduced disease transmission, often called social immunity^5^. The social immune response consists of specific actions across several individuals, which collectively protect the social group from infection^5,6^, including behavioral and physiological mechanisms, genetic and morphological defenses, and variation in spatial organization^5^. In social insects, which live in especially complex and interactive environments, the social immune response is highly adapted and specialized to different types of parasites and even different stages of infection within a colony^5,7–9^.

Social immune responses are well documented in honey bees (*Apis mellifera*) and are suspected to compensate for the reduced number of immunity-related genes found in honey bees compared to other sequenced insects^5,10,11^. Hygienic behaviors, typically observed as the selective removal of dead, damaged, or diseased brood, are an effective defense against bacterial infections and have been selected for in honey bee breeding programs^12^. Workers also collect and produce compounds with antimicrobial activity^13,14^ and can share immunological memory to the larvae^15^. Changes in behavior are also observed upon exposure to a parasite or pathogen, such as forced and altruistic removal from the colony^16^ and reduced social interactions^17^.

Throughout a larval honey bee’s development, it will receive nearly 10,000 visits consisting of cell inspections and provisioning by nurse bees^18^. This high amount of activity around a single larva, let alone the thousands of other larvae, provides an excellent opportunity for pathogens to spread through the oral secretions produced to feed larvae^19^. The ectoparasitic mite *Varroa destructor*, which vectors viruses such as Israeli acute paralysis virus (IAPV)^20–22^, also utilizes brood care behavior, using nurses as vehicles to introduce mites to new larval hosts^20,21,23,24^. Viruses can also be spread through the oral-gut pathway from workers to the brood. Deformed wing virus (DWV) has been detected in larval diets produced by infected worker bees and is infectious to larvae that consume contaminated food^25^. The transmission of IAPV through the larval diet is less studied in comparison to DWV but is likely to occur based on its detection in colony materials such as honey, pollen, and royal jelly^26^. Additionally, the hypopharyngeal glands, which synthesize the royal jelly that is fed to larvae, contain the third highest accumulation of IAPV particles after the gut and nerve tissue^26^.

IAPV infection in adults can occur through consuming contaminated food, a common method of experimental infection^17,27,28^, or through social interactions like trophallaxis^29^. Once adult bees are exposed, IAPV presents as a systemic infection, leading to shivering and often lethal paralysis^26,30^, although sublethal and asymptomatic infections have been observed as well^26^. Previously studied social immune responses to IAPV are not unlike responses to other pathogens; infected workers engage in fewer social behaviors such as trophallaxis^17^, and queens may have a preference (albeit not statistically significant) for interacting with uninfected workers as opposed to infected workers^29^. At the same time, however, these responses are context dependent, and IAPV infection can also result in behavioral changes that can increase virus transmission^17^, in other words, host manipulation^31^. For example, foragers experimentally infected with IAPV are more likely to be accepted into a foreign colony than uninfected bees, increasing pathogen transmission between colonies^17^.

Given the high potential for IAPV transmission through brood care and the highly adapted nature of the social immune response in honey bees, we tested the hypothesis that nurse bees exposed to IAPV exhibit a social immune response in an effort to reduce virus transmission. Under this social immunity hypothesis, we predicted that the frequency of virus infection in the social environment (e.g. the percentage of nurse bees exposed to IAPV in a given social group, or simply the percentage-exposed) influences the group’s larval care response, specifically that groups of workers with a higher percentage of exposed bees will reduce larval contact in order to avoid virus movement to larvae. Similarly, we predicted that, at the individual level, nurse bees exposed to IAPV will reduce their brood care behavior relative to uninfected counterparts when housed together in the same social environment. An alternative hypothesis is that IAPV manipulates the behavior of its host to increase transmission, as it can in some contexts^17^. Thus, under this host manipulation hypothesis, we predicted that IAPV exposure would lead to more contact with larvae to facilitate transmission.

## Materials and methods

### Cage set-up and experimental infection

The following methods were designed to generate experimentally infected and uninfected populations of age-matched honey bee workers that can be assayed when the prime age for observing nursing behavior coincides with the peak of IAPV infection: 7 days after emergence^32^ and 48 h after exposure to the virus^17,27,33^, respectively. Thus, newly emerged worker bees were sourced from three colonies across two days at the University of Illinois Urbana-Champaign Bee Research Facility (Urbana, IL 61801, USA) in late July 2022. Newly emerged bees were marked with one of ten colors using a paint pen (Sharpie) for individual recognition and placed in acrylic cube cages in groups of 35^28,34^ according to color marking. This process was repeated using the same ten colors, ultimately resulting in two sets of ten cages, each containing 35 newly emerged bees all marked with the same color. Cages were provisioned ad libitum with 30% sucrose solution via drip feeder and artificial protein supplement (MegaBee). Artificial protein supplement was used to limit potential variation in nutrition due to pollen diets or pathogen contamination present in natural pollens. Artificial supplement does not interfere with the development of the hypopharyngeal glands when compared to natural pollen diets^35^, so no effect on nursing behavior from the diet was expected. All cages were kept within a walk-in incubator kept at 34 °C and 50% relative humidity^28,32,34^.

Five days after the initial cage set-up and two days prior to behavioral observations, the sucrose feeders and protein supplements were removed from each cage. Each cage in one of the two sets of ten received a sublethal dose of IAPV, diluted in 600 µl of 30% sucrose solution (approximately 126,507 genome equivalents per cage, or 3614 genome equivalents per bee)^17,27,36^ for oral exposure. The other cages in each set received only 600 µl of 30% sucrose solution. After 24 h, the sucrose feeders and protein supplements were returned.

**Figure 1:**
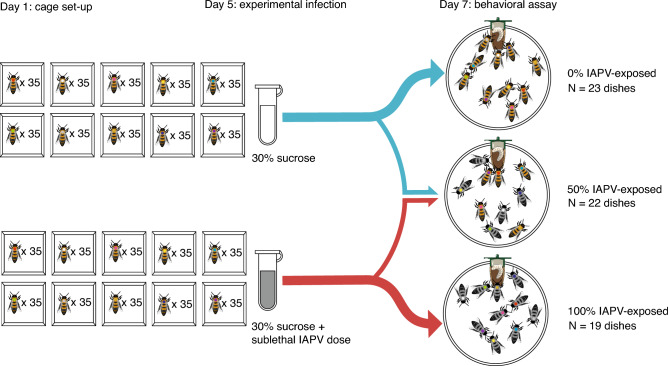
Visualization of cage and assay set-up. Day-old bees were placed in groups of 35 into two sets of ten acrylic cages. Each cage of bees received a different color-marking. After 5 days, one of the sets of ten cages was experimentally infected with a sublethal dose of IAPV diluted in sucrose. The other group received untreated sucrose. Two days after exposure, bees were transferred individually from the cages into the assay dishes, such that each dish had ten uniquely marked individuals. Bees were drawn from either the exposed cages or the unexposed cages in order to make the three different social environments: 0% of bees exposed, 50% of bees exposed, and 100% of bees exposed.

Behavioral observations were initiated two days after IAPV exposure, when IAPV levels within the adults are most likely to peak, as well as when previous work has observed behavioral differences due to covert infection^17,27,33^, and seven days after the initial cage set-up, when nursing behavior is best observed using this assay^32^. Prior to beginning the assay, one adult bee of each of the ten color markings was transferred from its respective cube cage into a vertically oriented Petri dish (100 × 20 mm) with a beeswax foundation sheet pressed against the base of the dish to mimic in-hive conditions^32^, ultimately resulting in ten uniquely marked individuals per dish. Adult bees were pulled either from the virus exposed cages or the strictly sucrose-fed cages in order to create three different social environments: dishes with 0% of individuals exposed to IAPV, dishes with 100% of individuals exposed, and dishes with 50% of individuals exposed (Fig. 1). Each dish was supplied with a 2.0 ml drip feeder containing 30% sucrose solution, inserted through a hole in the top of the dish. Following the dish assembly, all adult bees were left in the walk-in incubator with the lights on for 1 h before beginning the recording process.

Twenty-four larvae were grafted into commercial plastic queen rearing cups (JZ-BZ) over 2 days from a colony different to the colonies from where the workers were sourced. The larvae were then transferred and raised in a queenless colony, during which the colony would build a wax cell around the rearing cup (also referred to as a “queen cell”). Queen cells were removed from the colony four days after grafting^32^ for immediate use in the behavioral assay.

### Behavioral recordings

Dishes were recorded using a VIXIA HF R800 camcorder (Canon Inc.) in a haphazardly selected order. Prior to starting the recording, the number of dead bees in the assay dish, if any, were recorded (0% exposed: 1 dish with 1 dead bee; 50% exposed: 4 dishes with 1 dead bee, 1 dish with 2 dead bees; 100% exposed: 7 dishes with 1 dead bee, 1 dish with 2 dead bees). All deaths were observed to be due to aggression in the initial acclimation period. Upon starting the recording, the sucrose feeder was removed and replaced with a queen cell, after which the nurse bees’ interactions with the queen cell and each other were filmed. The recording was stopped 5 minutes after the queen cell was first inserted^32^. Queen cells with larvae were labeled individually for use per group-type and so larval identity could be recorded. On each recording day, the three experimental groups were each assigned a set of four larvae. The larvae were rotated between dishes in a haphazard order, such that the same larva would not be shared across different experimental groups (e.g. between the 50% and 0% exposed communities), and that the same larva was not used in an assay twice in a row. Recordings were uploaded to cloud data storage for later behavioral analysis. Following the recordings, all dishes and nurse bees were immediately frozen at − 80 °C for whole bee sample processing.

At the completion of the experiments, video recordings of the dishes were reviewed and analyzed. Behavioral interactions of interest included antennation around the opening of the queen cell (“external-antennation”) and inserting the head inside the queen cell (“insertion”), as both of these are indicative of the brood care response^32,37^. Upon observing one of these interactions, the color identity of the nurse, its associated treatment (exposed or unexposed), and the duration of the behavior were recorded to the nearest 1.0 s. The total number of interactions for each behavior and the number of unique nurses which entered the queen cell at least once (“responders”) were also recorded.

### Behavioral statistical analyses

For both types of recorded behaviors, the numbers of interactions were standardized by taking the respective sums for each dish and dividing by the number of living adult bees in the dish at the time of recording. Likewise, the same process was used to standardize the duration of interactions for both behaviors as well as the number of responders. The durations of external-antennation behaviors were log-transformed prior to statistical analysis. Analyses were conducted in R using the lme4 package^38^. Linear mixed models were constructed for the response variables (the number and duration of each interaction type as well as the mumber of responding workers) and using the percentage of bees exposed to IAPV as a fixed effect. The observer, date, and the identity of the larva used in the assay were used as random effects and included when model selection (based on AIC values) deemed it necessary. Model diagnoses were assessed using the performance package^39^. Final model specifics can be found in the Supplementary Material. Significance of the percentage-exposed effect was determined using likelihood ratio tests against the null models.

Within the 50% exposed dishes, the two treatments present were compared directly to each other for the same metrics that were used in the dish-level analysis (duration and number of external-antennation and insertion behaviors). Only responding workers (bees which interacted with the queen cell at least once, hereafter described as “nurses”) were used in these analyses (see Supplementary Tables S1, S2). This choice was made to limit the scope of our study to just nurse bees. Previous studies have used larval-response as a method of identifying nurse bees^40^. In single cohort groups, workers will begin differentiating their behaviors outside their normal temporal patterns^41^, thus, for example, some of the adults in our study may have been precocious foragers and would not have responded to the larva at all.

Generalized linear mixed models for external-antennation and insertion behaviors were constructed using the duration and number of interactions as the response variables and the treatment (either exposed or unexposed to IAPV) as a fixed effect. Poisson or negative binomial families were chosen based on AIC values, as were the inclusion of the following random effects: dish, date, observer, and larval identity^17^. Analyses were performed in R using the lme4 package^38^ and model diagnoses were assessed using the performance package^39^. Final model specifics can be found in the Supplementary Material. The significance of an individual nurse's treatment as a fixed effect was determined using likelihood ratio tests against the null models. When treatment proved to have a significant effect, estimated marginal means were used to compare the IAPV-exposed nurses to the unexposed nurses using the emmeans package^42^.

Similar analyses were used to compare the unexposed nurses between the 50% and 0% groups. As with the previous comparisons, only responding bees were used in the analysis (Tables S3, S4). Using the percentage-exposed as a fixed effect, the same list of random effects used in the prior individual level of analysis were either included or excluded based on AIC values. Final model specifics can be found in the Supplementary Material. The significance of the percentage-exposed effect was determined through likelihood ratio tests against the null models.

For all of the dish and individual behavioral responses, post hoc power analyses were conducted using the pwr package in R^43^ for an estimated medium effect (*d* = 0.50) and an alpha of 0.05. Plots were constructed in R with ggplot2^44^, followed by legend and color adjustments in Adobe Illustrator (Adobe Systems).

### Dry head mass and virus quantification

We investigated whether our treatments had an effect on the worker hypopharyngeal glands, as these organs are closely associated with nursing behavior and produce royal jelly. Dry head mass was used as a proxy for hypopharyngeal gland development, as dry mass has been used previously as a measurement for nutritional physiology^45^. Individual adult bees were selected via random number generator from the 50% infected group and then separated by treatment (*N* = 30 bees per treatment). Heads were removed from the rest of the body and placed in a drying oven (60 °C for 48 h) before removing the antennae and weighing to the nearest 0.1 mg^45^. Bodies were set aside for RNA extraction and subsequent virus quantification. Head masses between treatments were compared with a two-sample t-test in R.

To confirm whether the experimental infection was successful, a subset of workers of each treatment (exposed *N* = 15 bees; unexposed *N* = 14 bees) used in the dry head mass study were randomly selected using a random number generator. An additional randomly selected subset of workers from the 0% (*N* = 14 bees) and 100% (*N* = 15 bees) exposed groups were also included and decapitated. Whole body RNA was extracted using TRIzol Reagent (Invitrogen) and treated with DNase I (New England Biolabs) before diluting to 100 ng/µl, quantified via nanodrop. IAPV RNA was quantified from the whole body samples in triplicate with one step RT-qPCR using the Power SYBR Green RNA-to CT 1-Step Kit (Applied Biosystems), as performed in previous studies^28^. Previously established primers were selected for IAPV (Forward: TGCAAGTGAACGCCCCAAAAACG; Reverse: TGCCACAGTTCCGACAACATCTGC)^33^, and initial quantities were calculated using a serially diluted standard curve (1:10) of viral RNA^28,33,34^. Initial quantities were log-transformed prior to statistical analysis^46^. Differences in viral RNA quantities between treatments were determined in R using pairwise Wilcoxon rank sum tests, followed by a Benjamini–Hochberg adjustment. Figures for the virus quantification and head mass comparisons were created with ggplot2^44^ in R, followed by legend and color adjustments in Adobe Illustrator (Adobe Systems).

## Results

In total, we observed 64 unique social groups (or 64 unique dishes), each with one of three percentages of individuals exposed to IAPV (0% exposed *N* = 23 dishes, 50% exposed *N* = 22 dishes, 100% exposed *N* = 19 dishes). The whole social group’s average number and duration of external antennation and insertion behaviors were compared between the three percentages of infection, as well as the number of responding workers to the queen cell. For individual level behaviors within the social groups with 50% of individuals exposed to IAPV, only the responding workers for each type of behavior were included in the analysis (external-antennation: exposed *N* = 76 nurses, unexposed *N* = 81 nurses; insertion: exposed *N* = 42 nurses; unexposed *N* = 39 nurses). For these nurses, the average number and duration of both types of interaction were compared between the two treatments. For each behavioral comparison, post hoc power analyses confirmed that the experimental designs each achieved an estimated power greater than 0.80^43^.

### Group level behaviors

**Figure 2:**
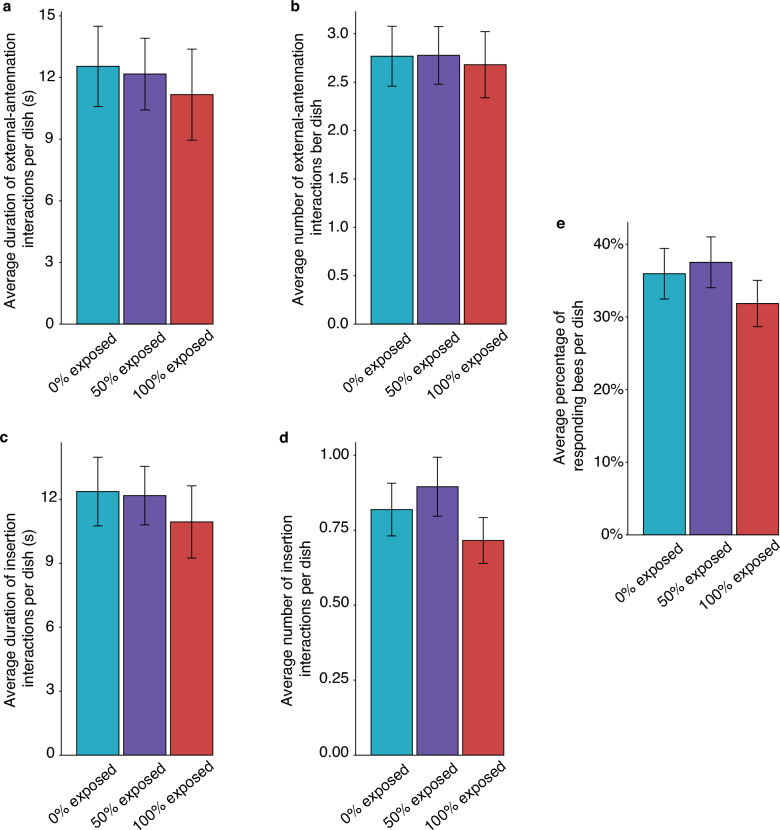
Response to the queen cell does not differ between three types of social group. Dishes containing workers with varying percentages of individuals exposed to IAPV were observed for 5 minutes. All group-level responses for a given dish were standardized by dividing by the number of living bees in the dish at the time of recording. The average duration (**a**) and number (**b**) of external-antennation interactions to the queen cell do not differ between the three types of social group (duration: LRT, *p* = 0.611; number: LRT, *p* = 0.938). The average duration (**c**) and number (**d**) of insertion-interactions do not differ between the three types of social group (duration: LRT, *p* = 0.575; number: LRT, *p* = 0.130). (**e**) The average percentage of bees that interacted with the queen cell at least once (responders) does not differ between the three types of social group (LRT, *p* = 0.273). Displayed values are means ± s.e.m.

Varying the percentage of bees exposed to IAPV within a social group does not have a significant effect on the whole group’s time spent antennating around the opening of the queen cell (Fig. 2a, likelihood ratio test against the null model: *X*^2^ = 0.984, *df* = 2, *p* = 0.611) or the number of external-antennation behaviors (Fig. 2b, likelihood ratio test against the null model: *X*^2^ = 0.128, *df* = 2, *p* = 0.938). Likewise, the percentage of IAPV-exposed workers had no significant effect on the duration of time spent inside the queen cell (Fig. 2c, likelihood ratio test against the null model: *X*^2^ = 1.11, *df* = 2, *p* = 0.575), or the number of insertion interactions (Fig. 2d, likelihood ratio test against the null model: *X*^2^ = 4.08, *df* = 2, *p* = 0.130). Similarly, the percentage of exposed workers had no significant effect on the number of responding individuals in each dish (Fig. 2e, likelihood ratio test against the null model: *X*^2^ = 2.59, *df* = 2, *p* = 0.273).

### Individual level behaviors in the 50% exposed group

**Figure 3:**
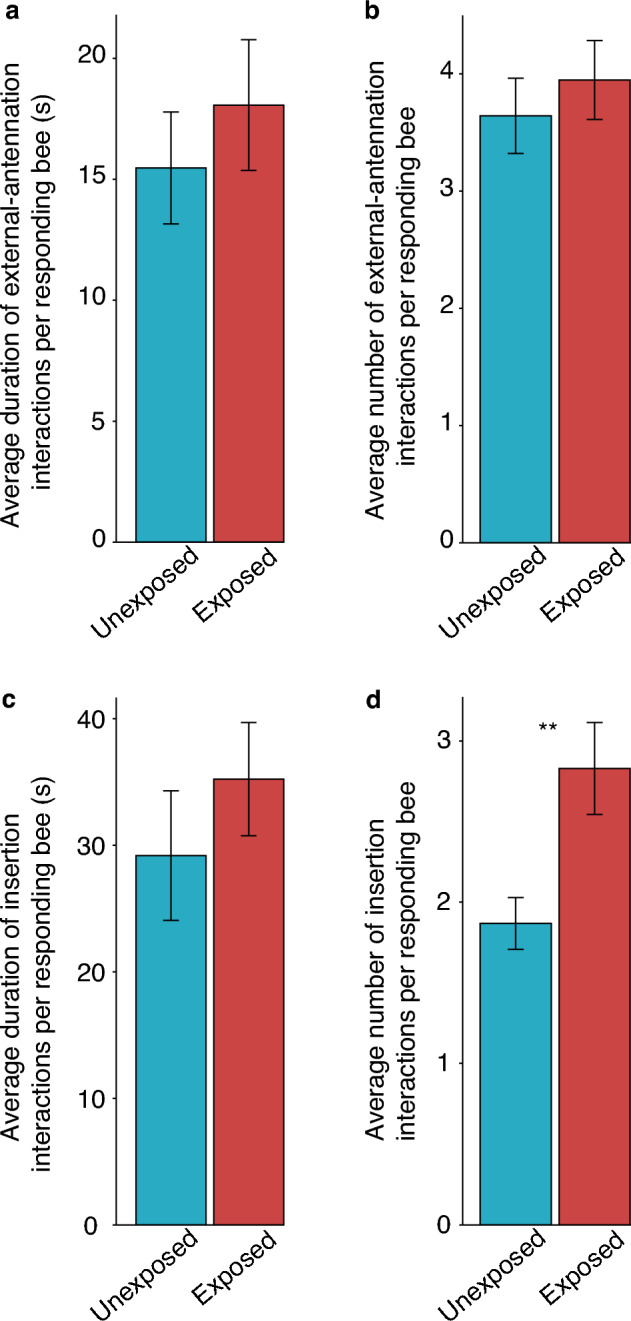
Average durations and counts of external-antennation and insertion-interactions per individual bee within the 50% exposed group. Nurse bees in dishes where 50% of the population were exposed to IAPV were observed for five minutes. Treatment (IAPV-exposed or unexposed) did not have a significant effect on the average total duration (**a**) and average number (**b**) of external-antennation interactions per bee (duration: LRT, *p* = 0.203; number: LRT, *p* = 0.367). (**c**) Treatment did not have an effect on the average total duration of insertion interactions (LRT, *p* = 0.337). (**d**) An individual bee’s treatment had a significant effect on the average number of insertion-interactions (LRT, *p* = 8.16 × 10^–3^**), and exposed bees entered inside the queen cell more often than unexposed bees (Tukey HSD, *p* = 8.7 × 10^–3^**). Displayed values are means ± s.e.m.

Similar to the analyses performed at the group level, we observed no significant effect of individual nurse treatment on the duration of external-antennation (Fig. 3a, likelihood ratio test against the null model: *X*^2^ = 1.62, *df* = 1, *p* = 0.203), the number of external-antennation interactions (Fig. 3b, likelihood ratio test against the null model: *X*^2^ = 0.814, *df* = 1, *p* = 0.367), or the duration of time spent inside the queen cell (Fig. 3c, likelihood ratio test against the null model: *X*^2^ = 0.921, *df* = 1, *p* = 0.337). However, treatment did have a significant effect on the number of insertion interactions (Fig. 3d, likelihood ratio test against the null model: *X*^2^ = 6.99, *df* = 1, *p* = 8.16 × 10^–3^**), with the IAPV exposed nurses entering the cell more than unexposed bees (estimated marginal means with Tukey HSD: *SE* = 0.222, *Z-ratio* = 2.63, *p* = 8.7 × 10^–3^**).

As we saw a difference in the number of insertion interactions between the exposed and unexposed bees in the 50% exposed environment, we questioned whether the unexposed bees were decreasing their level of attentiveness relative to completely unexposed environments as a means of avoiding self-contamination. We observed no differences in nursing attentiveness between the unexposed nurses from the 50% and 0% exposed groups. The presence of virus-exposed nurses did not have a significant effect on the duration of external-antennation (likelihood ratio test against the null model: *X*^2^ = 0.069, *df* = 1, *p* = 0.792) or the number of external-antennation interactions (likelihood ratio test against the null model: *X*^2^ = 0.0096, *df* = 1, *p* = 0.922). Likewise, no significant effect was observed on the duration of insertion interactions (likelihood ratio test against the null model: *X*^2^ = 0.828, *df* = 1, *p* = 0.363) or the number of insertion interactions (likelihood ratio test against the null model: *X*^2^ = 2.11, *df* = 1, *p* = 0.147).

### Dry head mass and virus quantification

There were no observed differences in dry head mass between the two types of treatment (two-sample *t* test: *t* = 0.210, *df* = 58, *p* = 0.835, Fig. S1), indicating that IAPV exposure 2 days prior to the behavioral observations did not cause anatomical differences that may affect the brood care response, such as the shrinking of hypopharyngeal glands^47^. Quantitative PCR for viral RNA confirmed that the adults in both the 50% and 100% exposed environments had significantly higher levels of IAPV transcripts relative to the unexposed adult bees in the 50% and 0% exposed environments (Table S5). The two types of exposed groups did not differ in viral titers, nor did the two unexposed groups (Fig. S2). The non-zero level of viral RNA detection in the uninfected bees can be attributed to regular background virus levels in the source colony^33^, and our detected levels are similar to the quantities found in untreated bees in other published studies on the effects of IAPV infection on behavior^17^.

## Discussion

Our results showed that exposure to IAPV did not affect overall group-level nursing responses; when comparing across group-level treatments where 100%, 50%, or 0% of nurses were exposed to IAPV, larvae always received the same amount of total care. However, while each larva received the same amount of attention no matter the makeup of nurse group, we observed differences in the nursing frequency between exposed and unexposed nurses within the groups that contained a mixture of control and IAPV-treated adult bees, with the IAPV-exposed nurses contacting larvae more frequently than controls.

Our experiment investigated two alternate hypotheses: first, that a social immune response will be present in the nurse bees’ response to a larva, and second, that IAPV manipulates host behavior to increase virus transmission. The uniformity in group-level responses does not clearly support either of our hypotheses but may be explained by trade-offs between a social immune response and an essential social behavior. For example, if avoidance behaviors are over-expressed, the larva may receive inadequate feeding and care^48^, resulting in detrimental effects on the colony’s health, as malnourished larvae are often culled^49,50^ or, if the larvae are reared to adulthood, develop into impaired adults^51^, ultimately acting as a less effective workforce^49,52^. These results are also likely not unique to IAPV. Similar responses have been observed when testing other bee pathogens and parasites, such as *Varroa destructor*^48^. Nurse bees, while under *Varroa* infestation, do not abandon the brood, despite nurse bees being the primary vehicle for *Varroa* dispersal^21,23,24^. This was accompanied, though, by a social immune response at the spatial organizational level; nurse bees were still observed near the brood, whereas foragers were found further away^48^. While our experiment created a controlled environment to observe nursing behavior, it is not a representative situation of a real colony, where cohort sizes are much higher than those used in our experimental arenas. It is possible that our experiment only captures a small slice of the social immune responses and, similar to the response towards *Varroa*^48^, an organizational response requiring a larger group size may also occur.

Within the group environment where 50% of nurses were exposed to IAPV, we found that IAPV-treated nurses interacted significantly more with larvae than controls. Additionally, we found no significant differences in the responses of the unexposed nurses in the 50% and 0% exposed groups, indicating that the unexposed bees in the 50% exposure group were not reducing their own responses as a means to limit self-contamination.

This result supports the hypothesis that IAPV infection results in behavioral manipulation of the nurse host that could spread the virus rather than a social immune response that could suppress transmission. Our investigation of dry head mass indicates that these differences were not due to anatomical differences in hypopharyngeal gland sizes which may influence nursing behavior^45,47^. While honey bees show social immune responses to virus infection, IAPV can also manipulate adult cuticular hydrocarbon (CHC) profiles and increase the likelihood that infected bees are accepted into foreign colonies, likely spreading infections^17^. Therefore, it is possible that IAPV may be altering the brood care-system in order to benefit its own transmission, as more frequent interactions with the larva may lead to a higher risk of viral transmission. If the observed increase in the number of interactions is due to IAPV, it is not unlikely that other parasites, such as *Varroa*, may exploit IAPV’s effect on brood care behavior. *Varroa*-parasitized bees may be more likely to be infected with IAPV^22^, thus increasing the likelihood that a *Varroa*-parasitized bee enters a brood cell and fulfilling the mite’s dispersal needs. IAPV benefits again from this effect on behavior, both from the direct transmission through oral secretions but also through increased vectored transmission. If this hypothesis is correct, future studies should incorporate *Varroa* parasitization and movement into their designs, as well as investigating whether *Varroa* prefer infected hosts to uninfected hosts.

While these findings seem to support only the hypothesis that IAPV is manipulating the host to increase transmission, there is another facet of social immunity that may instead be triggering the increased larval contact behaviors. Exposure to a parasite or pathogen promotes the bees’ natural hygienic response, during which honey bee workers inspect brood and remove dead, infected, or otherwise damaged individuals^53^. This is due to parasite-induced changes in the worker’s CHC profile, for example those caused by bacterial infection^54^, viral infection^17^, and *Varroa* parasitism^55^, which ultimately stimulate a hygienic response in the surrounding workers^54,56^; infection status can also induce self-grooming^57^. Because they have been stimulated by a sublethal virus infection, the experimentally infected workers in our experiments may be more alert to the presence of a pathogen within their environment than their unexposed nestmates, priming them to perform hygienic behaviors. These bees may then inspect the larva more frequently to determine if the larva also shows signs of infection. While our experiment was not designed to measure the worker’s hygiene thresholds, our data provide some circumstantial support for this hypothesis. In our comparison of exposed and control individuals, IAPV-treated nurses performed more “insertion” behaviors than controls – i.e. inserting their head into the cell to contact the larva. However, for an individual bee over the course of five minutes, the total time spent performing insertion behaviors did not differ between the exposed and unexposed nurses in the 50% exposed dishes. Thus, the higher number of visits performed by the exposed bees was likely accompanied by individually *shorter* visit times. Longer visits are more indicative of feeding events, during which the larva is presented food secretions, whereas shorter visits typically involve larval inspections or cell inspections and maintenance^18,32,58^. While it is well known that bees remove diseased or damaged brood^53^, to our knowledge, it is still unknown whether infected adults exhibit differences in their threshold or ability to detect or remove compromised larvae and pupae. Future work is necessary to understand how infection status of nurse bees affects hygienic responses at a colony level. This response may also have implications for parasite transmission; behaviors that are meant to stem the spread of certain parasites, such as allogrooming and hygienic behaviors, may instead facilitate transmission of others due to the increased number of intimate contacts^59,60^. However, whether this is host manipulation or simply the virus exploiting an existing behavior remains to be studied.

Using IAPV as a model virus for studying social immunity, we investigated how brood care, an essential behavior, is affected by viral exposure. However, further study with this system is required to determine whether the observed changes are supported by one or both of the discussed hypotheses, particularly in a larger social context that is more comparable to a full-sized colony. The group sizes used in this study are just one of the limitations. The assay design we used, as described by Shpigler & Robinson^32^, only records behaviors over the course of five minutes. It is possible that longer contact periods between the workers and the larvae may elicit a stronger response. Since the virus’ first description in 2007^30^, IAPV has been associated with colony losses^61^ and in many ways is not unlike an emerging infectious disease in other biological systems. Increased study on IAPV, especially its transmission through and interactions with host behavior, is imperative to better characterize the disease dynamics inside the highly specialized and interactive environment of a honey bee colony.

### Supplementary Information

Supplementary Information.

## Data Availability

The data supporting the findings of this study are openly available in “Dryad” at https://datadryad.org/stash/share/xoYSq34OktvhUNQC3140H0c4sejYWWrichzKRwyL_QA, https://doi.org/10.5061/dryad.k98sf7mcj.
