# Supplementary material for: The effect of Israeli acute paralysis virus infection on honey bee brood care behavior
Source: Sci Rep. 2024 Jan 10;14:991. doi: 10.1038/s41598-023-50585-4 (PMC10781695; doi:10.1038/s41598-023-50585-4)
Supplement: Supplementary file 1 — Supplementary Information. [file 41598_2023_50585_MOESM1_ESM.pdf]

The effect of Israeli acute paralysis virus infection on honey bee brood care behavior

*Lincoln N. Taylor[1] & Adam G. Dolezal[1]

[1]Department of Entomology, University of Illinois Urbana-Champaign, Urbana, IL 61801, USA

lnt3@illinois.edu

Department of Entomology, University of Illinois Urbana-Champaign

320 Morrill Hall, MC-118

505 S. Goodwin Ave.

Urbana, IL 61801-3750

(217) 333-2910

Supplementary Material

Supplementary Methods

Statistical analysis for the group level comparisons

Analyses for the group level comparisons were conducted in R using the lme4 package (Bates et al., 2015). Using the percentage of bees exposed to IAPV as a fixed effect, the final models for comparing the duration and number of external-antennation behaviors across the three levels of experimental IAPV infection included "observer" as a random effect. The duration of external-antennation was log-transformed prior to analysis. The final model for the duration of insertion behaviors included "larval identity" as a random effect. The final models for the number of insertion behaviors and the number of responders both included the "date" and "larval identity" as random effects. Model diagnoses were assessed using the performance package (Lüdecke et al., 2021). Significance of the percentage-exposed effect was determined using likelihood ratio tests against the null models.

Statistical analysis for the individual level comparisons

Analyses for the individual bee comparisons within the 50% exposed dishes used the same behavioral metrics that were used in the dish-level analysis (duration and number of external antennation and insertion behaviors). To determine if experimentally infected bees were more likely to interact at all with the queen cell, a contingency table was constructed comparing responding bees (bees that interacted with the queen cell at least once) to non-responding bees for both external-antennation and insertion behaviors, followed by a likelihood ratio test. Contingency table analyses were performed in JMP Pro 16. Because no significant differences between the treatments of the numbers of responding bees were observed for external-antennation behaviors (Supplementary Table S1, likelihood ratio: $N = 215$, $X^2 = 0.186$, $df = 1$, $p = 0.666$) or insertion behaviors (Supplementary Table S2, likelihood ratio: $N = 215$, $X^2 = 0.338$, $df = 1$, $p = 0.561$), non-responding bees were excluded from further analyses.

Using bee treatment as a fixed effect (IAPV-exposed or IAPV-unexposed), generalized linear mixed models were constructed in R using the lme4 package (Bates et al., 2015). The final models for the duration and number of external-antennation interactions both used negative binomial distributions and "dish" as a random effect. The final model for the duration of insertion

interactions used a negative binomial distribution and “larval identity” as a random effect, and the final model for the number of insertion interactions used a Poisson distribution and included “larval identity” as a random effect.

Similar to the analyses above, when comparing the unexposed bees in the 50% and 0% exposed groups, only the responding bees were used in the analysis. To verify this, contingency tables followed by likelihood ratio tests comparing response statuses between bees from the 50% or 0% exposed groups determined that the number of responding bees between the two dish environments were not significantly different from each other, and that the nonresponding bees could be excluded from further analysis for both insertion interactions (Supplementary Table S3, likelihood ratio test: $N = 338$, $X^2 < 0.001$, $df = 1$, $p = 0.996$) and external-antennation interactions (Supplementary Table S4**,** likelihood ratio test: $N = 338$, $X^2 = 1.121$, $df = 1$, $p = 0.290$).

Using the lme4 package in R (Bates et al., 2015), the percentage-exposed was used as the fixed effect, and the same list of random effects used in the prior individual level of analysis were included or excluded when AIC deemed necessary. The durations of external-antennation interactions were log-transformed, and a linear mixed model was constructed with dish as a random effect. The final model for the number of external-antennation interactions used a negative binomial distribution and included the dish and observer as random effects. A generalized linear model with a Poisson distribution was constructed for the number of insertion interactions, and a generalized linear mixed model following a Poisson-lognormal distribution was constructed for the duration of insertion interactions, including larval identity as a random effect.

Supplementary Table S1. *Number of responding and non-responding bees performing external-antennation across the IAPV-exposed and unexposed treatments.* Bees that antennated the queen cell at least once were designated as a "responder." Values represent the number of bees in each category. The numbers of responders and non-responders between infected and uninfected bees are not statistically different (likelihood ratio test: $N = 215$, $X^2 = 0.186$, $df = 1$, $p = 0.666$).

	**Non-responder**	**Responder**	**Total**
**Unexposed**	28	81	109
**Exposed**	30	76	106
**Total**	58	157	215

Supplementary Table S2. *Number of responding and non-responding bees performing insertion interactions across the IAPV-exposed and unexposed treatments.* Bees that entered inside the queen cell at least once were designated as a "responder." Values represent the number of bees in each category. The numbers of responders and non-responders between exposed and unexposed bees are not statistically different (likelihood ratio test: $N = 215$, $X^2 = 0.338$, $df = 1$, $p = 0.561$).

	**Non-responder**	**Responder**	**Total**
**Unexposed**	70	39	109
**Exposed**	64	42	106
**Total**	134	81	215

Supplementary Table S3. *Number of responding and non-responding unexposed bees performing insertion interactions across the 50% and 0% exposed dishes.* Bees that entered inside the queen cell at least once were designated as a "responder." Values represent the number of bees in each category. The numbers of responders and non-responders between infected and uninfected bees are not statistically different (likelihood ratio test: $N = 338$, $X^2 < 0.001$, $df = 1$, $p = 0.996$)).

	**Non-responder**	**Responder**	**Total**
**Unexposed bees from the 0% dishes**	147	82	229
**Unexposed bees from the 50% dishes**	70	39	109
**Total**	217	121	338

Supplementary Table S4. *Number of responding and non-responding unexposed bees performing external-antennation interactions across the 50% and 0% exposed dishes.* Bees that entered inside the queen cell at least once were designated as a "responder." Values represent the number of bees in each category. The numbers of responders and non-responders between infected and uninfected bees are not statistically different (likelihood ratio test: $N = 338$, $X^2 = 1.121$, $df = 1$, $p = 0.290$).

	**Non-responder**	**Responder**	**Total**
**Unexposed bees from the 0% dishes**	47	182	229
**Unexposed bees from the 50% dishes**	28	81	109
**Total**	75	263	338

Supplementary Table S5. *Summary of pairwise comparisons for IAPV quantification.* Exposed and unexposed bees from the three types of social environment were sampled for IAPV levels and compared using pairwise Wilcoxon rank sum tests. All displayed values use log-transformed data for the estimated genome equivalents per 200 ng of RNA.

**Treatment; percentage infected**	**Paired comparisons**	**Mean difference**	***S.E.* (±)**	***W***	***p* (*α* = 0.05)**
Unexposed; 0%	Unexposed; 50%	0.0161	0.0640	104	0.804
	Exposed; 50%	-0.752	0.179	24	2.66×10^{-4}***
	Exposed; 100%	-1.12	0.177	4	9.28×10^{-7}***
Unexposed; 50%	Unexposed; 0%	-0.0160	0.0640	104	0.804
	Exposed; 50%	-0.768	0.177	189	1.75×10^{-4}***
	Exposed; 100%	-1.13	0.180	207	9.28×10^{-7}***
Exposed; 50%	Unexposed 0%	0.752	0.179	24	2.66×10^{-4}***
	Unexposed; 50%	0.768	0.177	189	1.75×10^{-4}***
	Exposed; 100%	-0.367	0.880	127	0.680
Exposed; 100%	Unexposed; 0%	1.12	0.177	4	9.28×10^{-7}***
	Unexposed; 50%	1.13	0.180	207	9.28×10^{-7}***
	Exposed; 50%	0.367	0.880	127	0.680

p-values adjusted with a Benjamini-Hochberg correction.

***$p < 0.001$

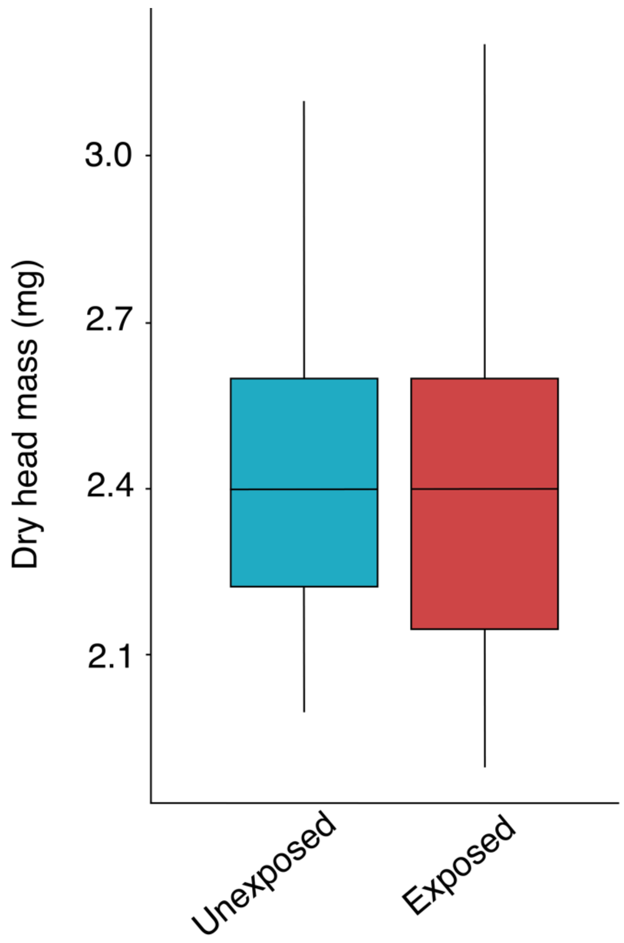

Supplementary Figure S1. *Dry head mass does not differ between exposed and unexposed bees*. Box plots show dry head mass (mg) for both exposed (N = 30 bees) and unexposed (N = 30 bees) bees pooled from all three social environments (two-sample t-test: t = 0.210, df = 58, p = 0.835).

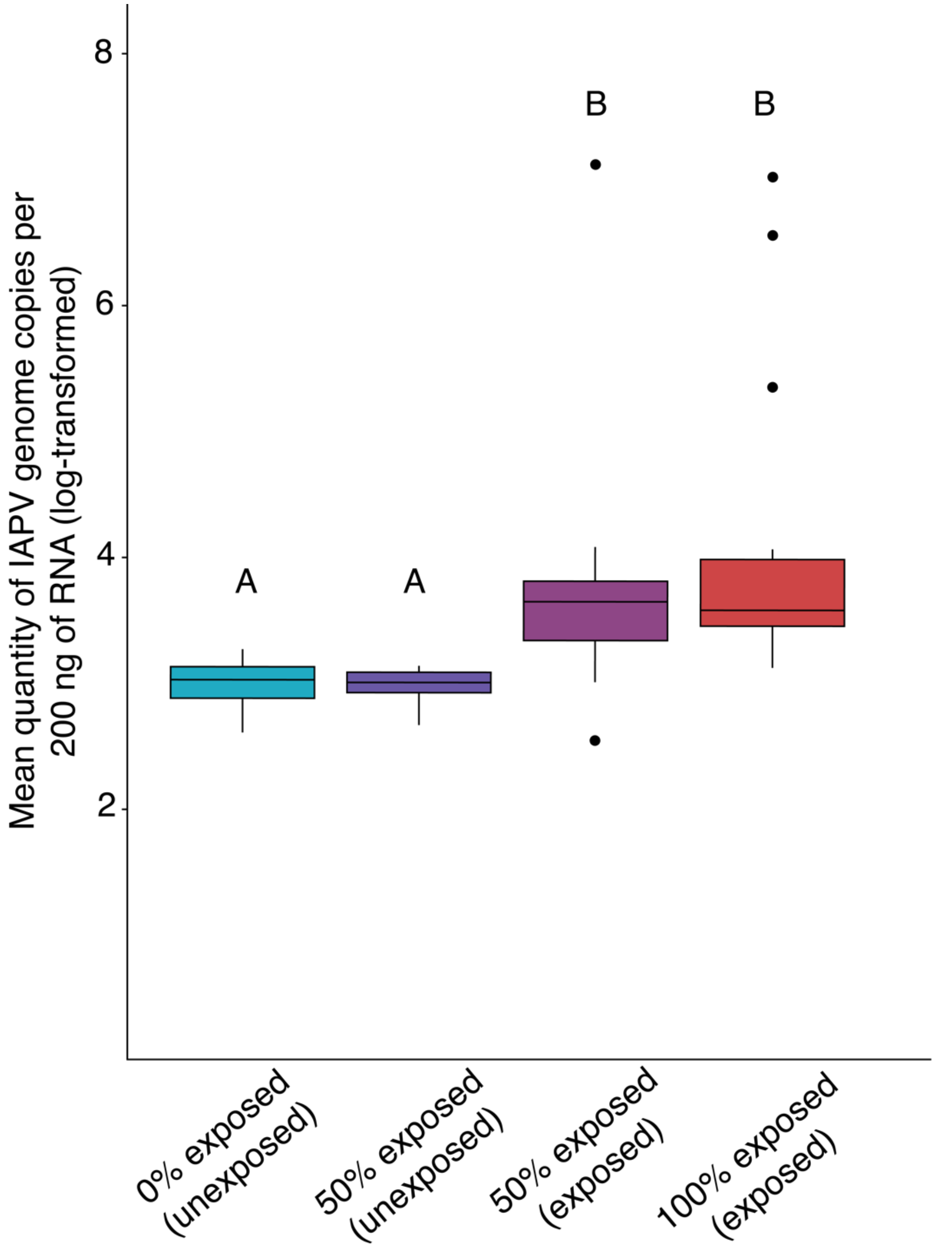

Supplementary Figure S2. *Validation of IAPV exposure treatment*. Box plots show the log-transformed quantities of IAPV genome copies per 200 ng of RNA for each bee treatment (exposed or unexposed) across the three different levels of group IAPV-exposure (0% exposed: $N = 14$ bees; 100% exposed: $N = 15$ bees; 50% exposed, exposed bees only: $N = 15$ bees; 50% exposed, unexposed bees only: $N = 14$ bees). Letters indicate statistically significant differences (pairwise Wilcoxon rank sum tests with Benjamini-Hochberg *p*-value adjustments).
